# Histone deacetylase HDAC2 regulates microRNA‐125a expression in neuroblastoma

**DOI:** 10.1002/brb3.2401

**Published:** 2022-01-21

**Authors:** Denghui Liu, Xianglian Tang, Zhao Huang, Jiabing Wen, Yuxiang Zhou

**Affiliations:** ^1^ Department of Pediatric Surgery Hunan Children's Hospital Changsha P.R. China

**Keywords:** HDAC2, histone modification, microRNA‐125a, neuroblastoma, PHOX2B

## Abstract

**Background:**

Neuroblastoma (NB) is an infrequent childhood malignancy of the peripheral sympathetic nervous system and is accountable for about 10% of pediatric tumors. microRNA (miR)‐125a has been implicated to serve as a tumor suppressor in various cancers. Herein, we set out to ascertain whether miR‐125a exerts antitumor effects in NB.

**Methods:**

Downregulated miRNAs were identified by miRNA microarray analysis of NB tissues and paracancerous tissues. The expression of miR‐125a in NB tissues and cells was detected by reverse transcription‐quantitative (RT‐q) PCR, followed by prognostic analysis. Gene Ontology (GO) enrichment analysis was performed on target genes of differentially expressed miRNAs. Cell proliferation, apoptosis, and differentiation were detected by cell counting kit‐8 (CCK‐8), Hoechst staining, immunofluorescence, and western blot. NB cells were injected into nude mice to detect tumorigenic, apoptotic, and differentiation activities in vivo. Dual‐luciferase assay and chromatin immunoprecipitation (ChIP) were carried out to verify the binding relationship between miR‐125a and PHOX2B or histone deacetylases 2 (HDAC2), respectively. Finally, rescue experiments were conducted.

**Results:**

miR‐125a was downregulated in NB tissues and cells, which was associated with poor prognosis. miR‐125a reduced NB cell proliferation and augmented apoptosis and differentiation. NB cells with miR‐125a overexpression decreased cell tumorigenesis and increased apoptosis and differentiation in xenograft tumor tissues. miR‐125a targeted PHOX2B, which was highly expressed in NB tissues and cells. HDAC2, highly expressed in NB tissues and cells, repressed miR‐125a transcription through histone deacetylation. Overexpression of HDAC2 or PHOX2B rescued the effects of miR‐125a on NB cell proliferation, apoptosis, and differentiation.

**Conclusion:**

HDAC2 inhibited miR‐125a transcription through deacetylation, and miR‐125a suppressed NB development through binding to PHOX2B.

## Graphical abstract

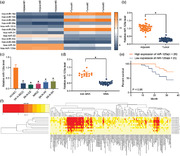

## 1 INTRODUCTION

Neuroblastoma (NB), the most common extracranial malignancy of childhood and the most common malignancy in infants, is an embryonal tumor of the sympathetic nervous system, developing during fetal or early postnatal time from sympathetic cells derived from the neural crest (Davidoff, 2012). NB is the major cause of death of children between the ages of 1 and 5 years and is responsible for about 13% of all mortality for pediatric cancers (Louis & Shohet, 2015). The most well‐known prognostic factors for NB are age, stage, and MYCN amplification, and more than half of children diagnosed with high‐risk NB either do not respond to conventional therapies or relapse after treatment (Berlanga et al., 2017). Therefore, understanding the molecular mechanism underlying NB is helpful to improve the prognosis of patients.

MicroRNAs (miRNAs) are small single‐stranded RNAs that target messenger RNAs (mRNAs) at the posttranscriptional level by inhibiting translation within all facets of human physiology, and miRNAs are currently used as biomarkers for prognosis and tumor characterization in multiple cancers, including NB (Zammit et al., 2018). In mammalians, miR‐125a is present in most adult organs and tissues where it targets proteins related to the mitogenic response, and the antiproliferative properties of miR‐125a, together with the fact that this miRNA is downregulated in many cancers, give a substantial support to the notion that miR‐125a exerts an onco‐suppressive effect (Russo & Potenza, 2019). Moreover, epigenetic mechanisms, mainly including DNA methylation, posttranslational histone modifications, as well as noncoding RNAs, tightly control gene expression, embryogenesis, and tumorigenesis, thus playing a vital role in both physio‐ and pathological settings (Vogelstein et al., 2013). Acetylation is one of the main posttranslational protein modifications with various effects on the protein level and the metabolome level, and the interplay between acetylation and deacetylation is of great importance for cellular processes (Drazic et al., 2016). The role of histone deacetylases (HDACs) has been defined by shedding light on the possible mechanisms involved in NB tumorigenesis, acting as potential markers and novel therapeutic options (Fetahu & Taschner‐Mandl, 2021). For one of them, HDAC2 was enhanced by MYCN, which in turn elevates stability and protein expression of MYCN, thereby describing a positive feedback loop (Kim & Carroll, 2004). Interestingly, HDAC2 has been reported to promote the development of tamoxifen resistance in breast cancer cells by downregulating miR‐125a‐5p (Huang et al., 2017). However, the possible correlation between HDAC2 and miR‐125a remains unclear in NB. In this study, we sought to identify and functionally characterize the role of miR‐125a in NB, linking its downregulation to HDAC2.

## 2 MATERIALS AND METHODS

### 2.1 Subjects

The study was approved by the ethical committee of the Hunan Children's Hospital and was in accordance with 2011 Declaration of Helsinki. Written informed consent was obtained from guardians from all subjects prior to enrollment. In this study, cancerous tissues and adjacent tissues were collected from 43 NB patients from January 2015 to January 2018. The adjacent tissues were matched noncancerous tissue samples, which were collected from a segment of the resected specimen more than 5 cm away from the primary NB tissue site. The tissues from patients were stored at −80°C for RNA extraction. The clinical characteristics of the subjects are listed in Table 1.

**TABLE 1:** Clinical characteristics of neuroblastoma (NB) patients

Clinicopathologic features	Number (*n* = 43)
Age (years)	
≥2.5	23
<2.5	20
Gender	
Male	29
Female	14
INSS stage	
I‐II	25
III‐IV	18
MYCN	
Amplified	28
Nonamplified	15

Abbreviation: INSS, International Neuroblastoma Staging System.

### 2.2 miRNA microarray analysis

Tumor and adjacent tissues from three patients were randomly selected for gene expression analysis, and RNA was extracted using TRIzol reagent (Thermo Fisher Scientific Inc., Waltham, MA, USA). The isolated RNA was reverse transcribed into complementary DNA (cDNA) using the Superscript Reverse Transcriptase Kit (Transgene Biotech, Beijing, China). The cDNA was then hybridized with Human miRNA Expression Microarray V4.0 (Arraystar Inc., Rockville, MD, USA) and GeneChip Human Gene 1.0 ST Array (Thermo Fisher Scientific). The hybridization microarray was incubated with DNA in an incubator for 24 h. Gene expression data were acquired using the GeneChip Scanner 3000 7G system (Thermo Fisher Scientific) and analyzed using R software (R‐project). Expression data were normalized and quality controlled using Affy (Bioconductor, Seattle, WA, USA). Differentially expressed miRNAs were screened using Pheapmap (Bioconductor) at |Log2 FoldChange| >2, *p* < .01 and the heatmap was plotted.

### 2.3 Reverse transcription‐quantitative PCR

Total RNA was isolated from cells using the PureLink RNA Mini Kit (Life Technologies, Carlsbad, CA, USA) according to the manufacturer's instructions. The mRNA reverse transcription was conducted using the iScript cDNA synthesis kit (Bio‐Rad Laboratories, Hercules, CA, USA), or the mature miR reverse transcription was accomplished using the TaqMan miR Reverse Transcription Kit (Applied Biosystems, Inc., Foster City, CA, USA). ABI StepOne Real‐Time PCR System (Applied Biosystems) and SYBR Green Mix (Bio‐Rad) were utilized for mRNA expression, or TaqMan Universal Fast PCR Master Mix for miRNA expression. Relative mRNA and miRNA expression was examined using the 2^−∆∆^ *^Cq^* method and normalized to the levels of glyceraldehyde‐3‐phosphate dehydrogenase (GAPDH) and U6, respectively. The primers were synthesized by Sangon (Shanghai, China): miR‐125a, forward 5′‐CGGTCCCTGAGACCCTTTAAC‐3′, reverse 5′‐GTGCAGGGTCCGAGGT‐3′; HDAC2, forward 5′‐TAAATCCAAGGACAACAGTGG‐3′, reverse 5′‐GGTGAGACTGTCAAATTCAGG‐3′; PHOX2B forward 5′‐AGTGGCTTCCAGTATAACCCG‐3′, reverse 5′‐GGTCCGTGAAGAGTTTGTAAGG‐3′; MYCN forward 5′‐ACCCGGACGAAGATGACTTCT‐3′, reverse 5′‐CAGCTCGTTCTCAAGCAGCAT‐3′; U6 forward 5′‐CTCGCTTCGGCAGCACA‐3′, reverse 5′‐AACGCTTCACGAATTTGCGT‐3′; and GAPDH forward 5′‐GATTCCACCCATGGCHDAC2TTC‐3′, reverse 5′‐AGCATCGCCCCACTTGATT‐3′.

### 2.4 Cell lines

Human NB cell lines SK‐N‐BE(2), SK‐N‐DZ, IMR32, SK‐N‐AS, and SK‐N‐SH were from American Type Culture Collection (Manassas, VA, USA), human umbilical vascular endothelial cells (HUVEC) were from the Cell Bank of Shanghai Institute of Cells, Chinese Academy of Science (Shanghai, China), and HEK 293T cells were from Thermo Fisher Scientific. Cells were cultured in Dulbecco's modified Eagle's/F12 medium (Corning Inc., Corning, NY, USA) with 10% fetal bovine serum (FBS) (Atlas Biologicals, Fort County, CO, USA).

### 2.5 Transfection procedures

miR‐125a mimic, HDAC2‐overexpression (OE), PHOX2B‐OE, and negative control oligonucleotides were purchased from Dharmacon (Lafayette, CO, USA). The cells were transiently transfected using Lipofectamine 2000 (Thermo Fisher) according to the instructions, and then cultured until they were stable. The cells were plated in 24‐well plates at 2000 cells/well, and HDAC2 inhibitor BRD5298, HDAC6 inhibitor BRD8148, and HDAC8 inhibitor PCI‐34051 (Broad Institute Chemical Biology Platform, Cambridge, MA, USA) were added in triplicate to each well at a final concentration of 10 μM. A same dose of dimethyl sulfoxide (DMSO) was chosen as the negative control.

### 2.6 Bioinformatics analysis

Differentially expressed genes‐mediated biological processes in NB were analyzed using by Gene Ontology (GO) enrichment analysis. GO enrichment analysis of miRNA target genes was performed in DIANA‐miRPath v3.0 (http://snf‐515788.vm.okeanos.grnet.gr/). GO enrichment analysis of differentially expressed genes in NB tissues was performed using the ClusterProfiler package (Bioconductor) and visualized by the Barplot package (version 3.6.3, R). Annotation information of GO biological pathway was downloaded from the GO database (http://www.bioconductor.org/packages/release/data/annotation/). Differentially expressed mRNAs were obtained from the GSE25624 data file in the Gene Expression Omnibus (GEO) database, and the LIMMA package described in Bioconductor (version 3.6.3, R) was used to determine gene expression between cancer and normal samples. A volcanic map was plotted using the volcano package (version 3.6.3, R).

### 2.7 Cell counting kit‐8

Cell counting kit‐8 (CCK‐8) analysis was performed according to the manufacturer's instructions (Dojindo Molecular Technologies, Kumamoto, Japan). The transfected cells were plated in 96‐well plates at 37°C with 5% CO_2_. After 24 h, transfected cells were plated again into 96‐well plates at 3 × 10^3^ cells/well and incubated stably for 96 h before adding 10 μl CCK‐8 solution and incubated for another 1 h. The optical density (OD) value at 450 nm was measured with a microplate reader.

### 2.8 Hoechst staining

After 96 h of transfection, the cells were plated into 24‐well plates, fixed with 0.5 ml fixative for 10 min, stained with 0.05 μg/ml Hoechst 33258 (Beyotime, Shanghai, China), and then placed on a shaker for 5 min. Fluorescence microscopy (Nikon Instruments Inc., Melville, NY, USA) can detect blue colored nuclei with an excitation wavelength of about 350 nm and an emission wavelength of about 460 nm. Cells with uniform chromatin staining were considered healthy, while cells with fragmented or condensed chromatin were considered apoptotic.

### 2.9 Immunofluorescence

Cells were fixed with 4% paraformaldehyde and 0.1% Triton X‐100 in phosphate‐buffered saline (PBS) buffer for 20 min. Fixed cells were incubated for 1 h at room temperature in a blocking solution (PBS containing 0.5% FBS) to reduce nonspecific binding. Cells were treated with diluted primary antibody in the blocking solution overnight at 4°C, and with diluted secondary antibody in the blocking solution for 1 h at room temperature. The monoclonal antibody β‐Tubullin III (ab52623, RRID: AB 869991, 1:500; Abcam, Cambridge, MA, USA) was used as the primary antibody and Alexa Fluor‐conjugated IgG (ab150077, RRID: AB 2630356, 1:1,000; Abcam) as the secondary antibody. 4′,6‐diamidino‐2‐phenylindole was used to stain cell nuclei (blue) for 30 min at a concentration of 1.43 μM, and cells were imaged with a fluorescent microscope (Nikon).

### 2.10 Western blot

Cells were lysed in radio immunoprecipitation assay buffer (150 mM NaCl, 1% NP‐40, 0.1% sodium dodecyl sulfate [SDS], 50 mM Tris [pH = 8.0]) supplemented with 1 μg/ml peptidase, 1 mM dithiothreitol, 0.2 mM phenylmethanesulfonyl fluoride, and 1 μg/ml pepsin inhibitor A. Concentrations were determined by the Bradford protein assay (BioRad). Equal amounts of proteins were separated by SDS‐gel electrophoresis and electro‐transferred to nitrocellulose membranes. The membranes were sealed with 0.5% skimmed milk for 60 min and probed with primary antibodies to neuron specific enolase (NSE, sc‐271384, RRID: AB_10609119, 1:2000; Santa Cruz Biotechnology Inc., Santa Cruz, CA, USA), growth associated protein 43 (GAP43, RRID: AB_627660; sc‐17790, 1:1,000; Santa Cruz), Bax (sc‐7480, RRID: AB_626729, 1:3000; Santa Cruz), cleaved‐caspase‐3 (NB100‐56708, RRID: AB_837846, 1:1,000; Novus Biological Inc., Littleton, CO, USA), HDAC2 (ab12169, RRID: AB_2118547, 1:1,500; Abcam), PHOX2B (sc‐376997, RRID: AB_2813765, 1:2,000; Santa Cruz), and GAPDH (AM4300, RRID: AB_2536381, 1:5,000; Thermo Fisher) overnight at 4°C. The membranes were then incubated with horseradish peroxidase‐labeled secondary antibody (ab205719, RRID: AB_2755049, 1:5,000; Abcam) for 60 min at room temperature. All membranes were examined using Western Lightning Plus‐ECL (Perkin Elmer) and analyzed using LAS‐3000 (FUJIFILM Wako Pure Chemical Corporation, Osaka, Japan) to assess relative bands and molecular weights relative to standard molecular weight markers.

### 2.11 Xenograft mouse model

Animal experiments were approved by the institutional animal care and use committee of the Hunan Children's Hospital. All experiments conform to the relevant regulatory standards. Thirty‐six‐week‐old BclB/C mice were purchased from Beijing Vital River Laboratory Animal Technology Co., Ltd. (Beijing, China) and inoculated with stably transfected 4 × 10^6^ SK‐N‐DZ and SK‐N‐AS cells (100 μl) at the left forelimb costal abdomen (*n* = 5). All mice were monitored daily to ensure the health of the injection site, and tumor volumes were measured weekly. Tumor volume was calculated by length × width^2^. Mice were executed by intraperitoneal injection of 1% pentobarbital sodium (120 mg/kg) on day 28, and tumors were euthanized to measure tumor weight. A portion of the excised tumors were used for immunohistochemistry and the remaining for western blot.

### 2.12 Immunohistochemistry

The mouse xenograft tumors were fixed in paraformaldehyde for 1 h, followed by conventional paraffin‐embedding and sectioning (4 μm), and drying at 60℃. Paraffin sections were dewaxed with xylene and graded ethanol, and rinsed with distilled water. The slides were boiled in sodium citrate buffer (pH = 6.0, Solarbio, Beijing, China) for 10 min for antigen recovery. The sections were incubated in 3% hydrogen peroxide (Solarbio) for 10 min to block endogenous peroxidase activity and in 5% normal goat serum (ZGB‐BIO, Beijing, China) to block nonspecific protein binding sites. The sections were then incubated with antibodies against cleaved‐caspase‐3 (1:500; NB100‐56113, Novus Biologicals) and β‐Tubulin‐III (ab52623, RRID: AB_2819160, 1:800; Abcam) overnight at 4°C and subsequently incubated with goat antirabbit biotin‐conjugated secondary antibody (ab205718, 1:800; Abcam) for 0.5 h at room temperature. The reaction products were characterized using diaminobenzidine (Sigma‐Aldrich Chemical Company, St Louis, MO, USA). Nuclei were counted lightly with hematoxylin (Solarbio), and immunostaining was observed under a light microscopy (Olympus Optical Co., Ltd., Tokyo, Japan). The mean OD values of different sections were compared by Image‐pro Plus software.

### 2.13 Luciferase reporter assay

StarBase (http://starbase.sysu.edu.cn/) was utilized for predicting potential binding sites between miR‐125a and the 3′UTR of PHOX2B mRNA. The predicted miR‐125a binding site in the 3′UTR of PHOX2B mRNA (called WT) and the mutated site (called MT) were cloned into the luciferase vector pEZX‐MT06 (Genecopoeia, Rockville, MD, USA), and the empty pEZX‐MT06‐luciferase vector was used as a negative control. HEK293T cells were then cotransfected with luciferase reporter plasmid and miR‐125a mimic/miR‐125a control. Cells were lysed 48 h later, and luciferase activity of firefly and Renilla luciferase was assessed using the Dual‐Luciferase Reporter Assay protocol in the reagent (Promega Corporation, Madison, WI, USA) and a GloMax Multi+ luminometer (Promega). Renilla luciferase activity was normalized by firefly luciferase activity and expressed as a foldchange relative to the negative control value (set to 1).

### 2.14 Chromatin immunoprecipitation

The promoter of miR‐125a was obtained from UCSC (https://genome.ucsc.edu/index.html). Cells were lysed in a buffer containing 50 mM Tris‐HCl (pH = 8.1), 1% SDS, 10 mM ethylenediaminetetraacetic acid, and a complete protease inhibitor mixture (Roche Applied Science, Mannheim, Germany), followed by sonication to obtain 200–1000 bp DNA fragments. Chromatin immunoprecipitation (ChIP) was conducted following the protocol provided by a ChIP Assay Kit (Millipore Corp, Billerica, MA, USA) using antibodies against histone H4 acetylation (ac, ab109463; RRID: AB_10858987, Abcam), HDAC2 (sc‐81599; RRID: AB_2118560, Santa Cruz), and normal mouse IgG (sc‐2025, RRID: AB_737182, Santa Cruz). The cells were centrifuged at 10,000 *g* for 10 min at 4°C to remove insoluble material. Next, the cells were mixed with 900 μl ChIP dilution buffer and 20 μl 50× protease inhibitor cocktail, 60 μl ProteinA Agarose for 60 min at 4°C. The mixture was then incubated with antibodies overnight at 4°C, followed by washing with 60 μl ProteinA Agarose and 250 μl eluate. The cross‐linking was reversed with 20 μl 5 M NaCl. After elution with 500 μl eluent, the samples were incubated with 1 μl RNaseA at 37°C for 1 h, and DNA fragments were recovered for PCR analysis.

### 2.15 Statistics

The SPSS 22.0 software (IBM SPSS Statistics, Chicago, IL, USA) was used to carry out statistical analysis with two‐tailed *p* < .05 as a level of statistical significance. For statistical comparisons, the paired *t*‐test, a one‐way analysis of variance (ANOVA), and two‐way ANOVA with Tukey's test were performed as required. In all cases, error bars are representative of the standard deviation of the mean of three biological experiments unless otherwise stated.

## 3 RESULTS

### 3.1 miR‐125a is significantly poorly expressed in NB

**FIGURE 1:**
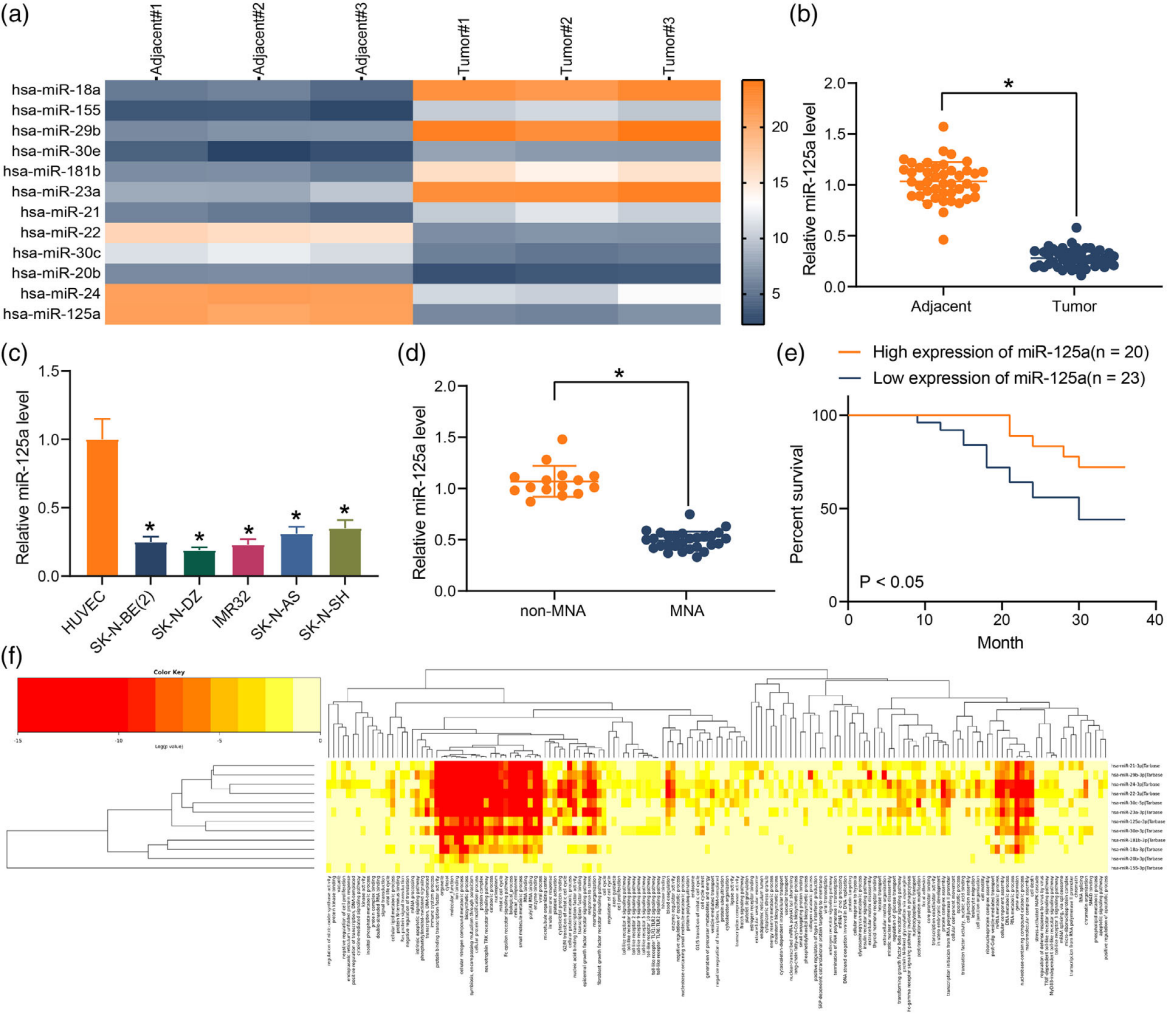
Relative expression of miR‐125a in neuroblastoma (NB) cell lines. (a) Differentially expressed miRNAs examined using microarray analysis. (b) miR‐125a expression between NB and normal tissues by reverse transcription‐quantitative (RT‐q) PCR. (c) miR‐125a expression between NB cells and human umbilical vascular endothelial cell (HUVEC) by RT‐qPCR. (d) Expression of miR‐125a in MYCN‐amplified (MNA) and non‐MNA NB tissues by RT‐qPCR. (e) Kaplan–Meier analysis of survival of patients differentially expressing miR‐125a. (f) Gene Ontology (GO) enrichment analysis of differentially expressed miRNAs‐enriched biological processes. Results are shown as mean ± SD, representative of three technical replicates (*p < .05 vs. adjacent tissues, HUVEC, or non‐MNA tissues by paired *t*‐test for panels B and D and by one‐way analysis of variance [ANOVA] for panel C)

Microarray analysis was performed using miRNA probes to identify miRNAs with tumor suppressor functions in NB, and miR‐125a was identified to be significantly reduced in NB (Figure 1a). To probe the function of miR‐125a in NB, we tested its expression in NB tissues. The data presented that miR‐125a was remarkably downregulated in NB tissues (*n* = 43) compared with adjacent tissues, which was the same as the microarray results (Figure 1b). Further analysis revealed that miR‐125a was significantly reduced in NB cell lines SK‐N‐BE(2), SK‐N‐DZ, IMR32, SK‐N‐AS, and SK‐N‐SH relative to HUVEC. Moreover, the downregulation was more pronounced in MYCN‐amplified cell lines SK‐N‐BE(2), SK ‐N‐DZ, and IMR32 (Figure 1c). The correlation between miR‐125a and clinical characteristics was analyzed in NB tissue samples, and miR‐125a expression was found to be much lower in MYCN‐amplified (MNA) patients (Figure 1d). Patients with low miR‐125a expression had shorter survival in the prognostic analysis (Figure 1e), demonstrating that low miR‐125a expression was closely linked to poor patient prognosis. The GO enrichment analysis of the differentially expressed miRNAs revealed that the differentially expressed miRNAs in NB were mainly enriched in apoptosis and cell differentiation processes, and miR‐125a was highly enriched in these processes (Figure 1f), indicating that miR‐125a mainly regulates NB cell activity through these pathways.

### 3.2 miR‐125a reduces activity in NB cell lines

**FIGURE 2:**
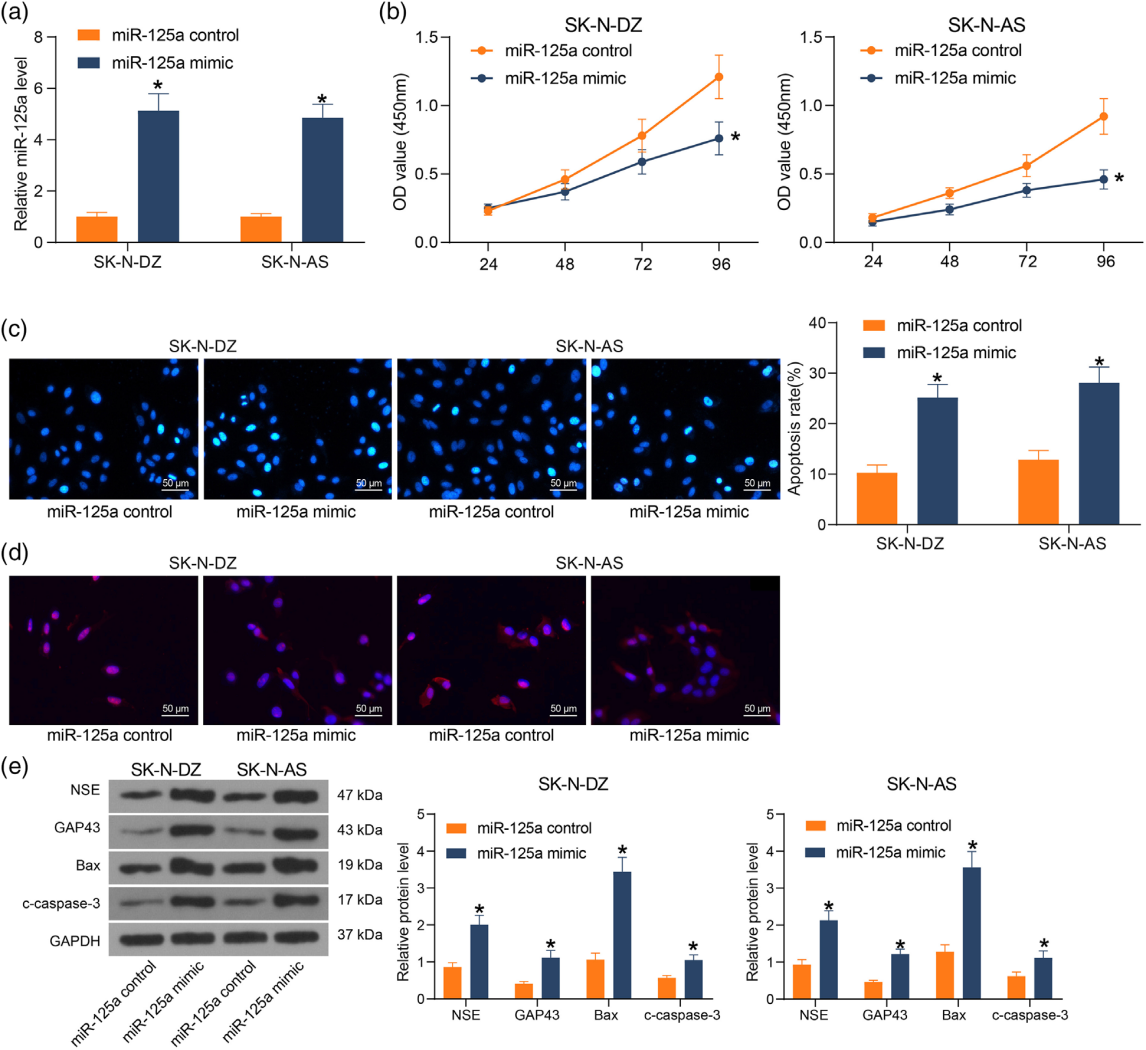
miR‐125a induces neuroblastoma (NB) cell apoptosis and differentiation in vitro. NB cell lines were transfected with miR‐125a mimic or miR‐125a control. (a) miR‐125a expression in cells after transfection measured using reverse transcription‐quantitative (RT‐q) PCR. (b) Cell proliferation examined using cell counting kit‐8 (CCK8). (c) Cell apoptosis assessed by Hoechst staining. (d) Immunofluorescence detection of β‐Tubulin‐III expression in cells to assess cell differentiation. (e) Protein expression of GAP43, NSE, Bax, and cleaved‐caspase‐3 in cells examined using western blot. Results are shown as mean ± SD, representative of three technical replicates (*p < .05 vs. miR‐125a control by two‐way analysis of variance [ANOVA])

The effect of miR‐125a on NB cell activity was examined according to the results of Figure 1f. The expression of miR‐125a was upregulated in MYCN‐amplified cell line SK‐N‐DZ and non‐MYCN‐amplified cell line SK‐N‐AS, and the success of transfection was RT‐qPCR (Figure 2a). We then investigated the cellular response to aberrant miR‐125a overexpression in NB cells. miR‐125a significantly reduced the OD value and cell proliferation (Figure 2b). To clarify whether the decrease in cell viability upon miR‐125a overexpression was due to the induction of cell death, the chromatin state of cells transfected with miR‐125a was examined by Hoechst staining. Cells transfected with miR‐125a showed a high percentage of chromatin condensation and fragmentation staining, which is one of the typical signs of apoptosis (Figure 2c). To observe the impact of miR‐125a on the differentiation and morphological changes of NB cells, we stained the cells with β‐Tubulin‐III to compare the cell differentiation upon treatment with miR‐125a mimic miR‐125 control. In the miR‐125a control‐treated cells, the cells were rounded and weakly differentiated, and β‐Tubulin‐III was concentrated in the cell body. On the contrary, in the miR‐125a overexpressing cells, the shape of undifferentiated cells changed to neuron‐like, the protrusions and neutral junctions were significantly prolonged, and β‐Tubulin‐III was expanded (Figure 2d). Further detection of apoptosis and cell differentiation via protein quantification revealed that miR‐125a increased the expression of pro‐apoptosis markers Bax and cleaved‐caspase‐3, as well as neuronal differentiation markers GAP43 and NSE in the cells (Figure 2e). The results showed that both apoptosis and differentiation may be responsible for the reduction of NB cell activity by miR‐125a overexpression.

### 3.3 miR‐125a reduces the proliferation of NB cells in vivo

**FIGURE 3:**
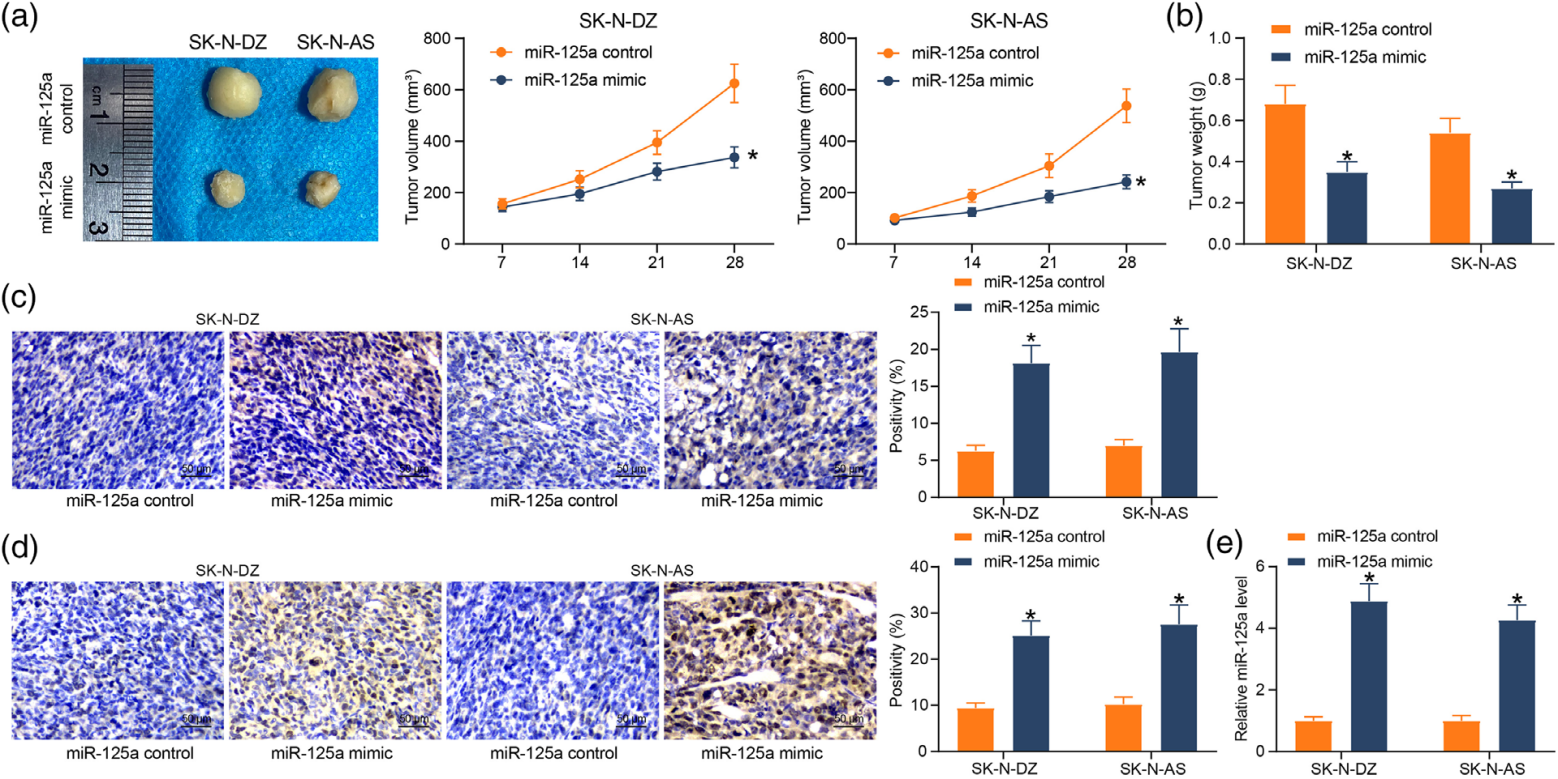
miR‐125a suppresses the proliferation of neuroblastoma (NB) cells in vivo. Nude mice were injected with stably transfected NB cells. (a) Tumor growth curve after injection of miR‐125a overexpressing cells. (b) Assessment of tumorigenic activity of cells by tumor weight. (c) Immunohistochemical detection of changes in cleaved‐caspase‐3 levels in tumor tissues. (d) Immunohistochemical detection of changes in β‐Tubulin‐III levels in tumor tissues. (e) miR‐125a expression in NB tissues by reverse transcription‐quantitative (RT‐q) PCR. Results are shown as mean ± SD, representative of three technical replicates (*p < .05 vs. miR‐125a control by two‐way analysis of variance [ANOVA])

We verified the ability of miR‐125a to reduce tumor growth in vivo by subcutaneous injection of SK‐N‐DZ and SK‐N‐AS cells previously transfected with miR‐125a mimic, and tumor growth was monitored every 7 days. Overexpression of miR‐125a delayed the growth of NB cells in vivo, resulting in a reduction of xenograft tumor volume and growth rate in mice (Figure 3a). Also, analysis of tumor weights confirmed the inhibition of xenograft tumors by miR‐125a overexpression (Figure 3b). The detection of cleaved‐caspase‐3 and β‐Tubulin‐III in tumors revealed that miR‐125a increased apoptosis and differentiation (Figure 3c,d). Verification of miR‐125a expression in tumors displayed that miR‐125a was consistently expressed in tumors (Figure 3e), indicating that miR‐125a overexpression reduced tumorigenic activity in NB cells and induced apoptosis and cell differentiation.

### 3.4 miR‐125a targets PHOX2B

**FIGURE 4:**
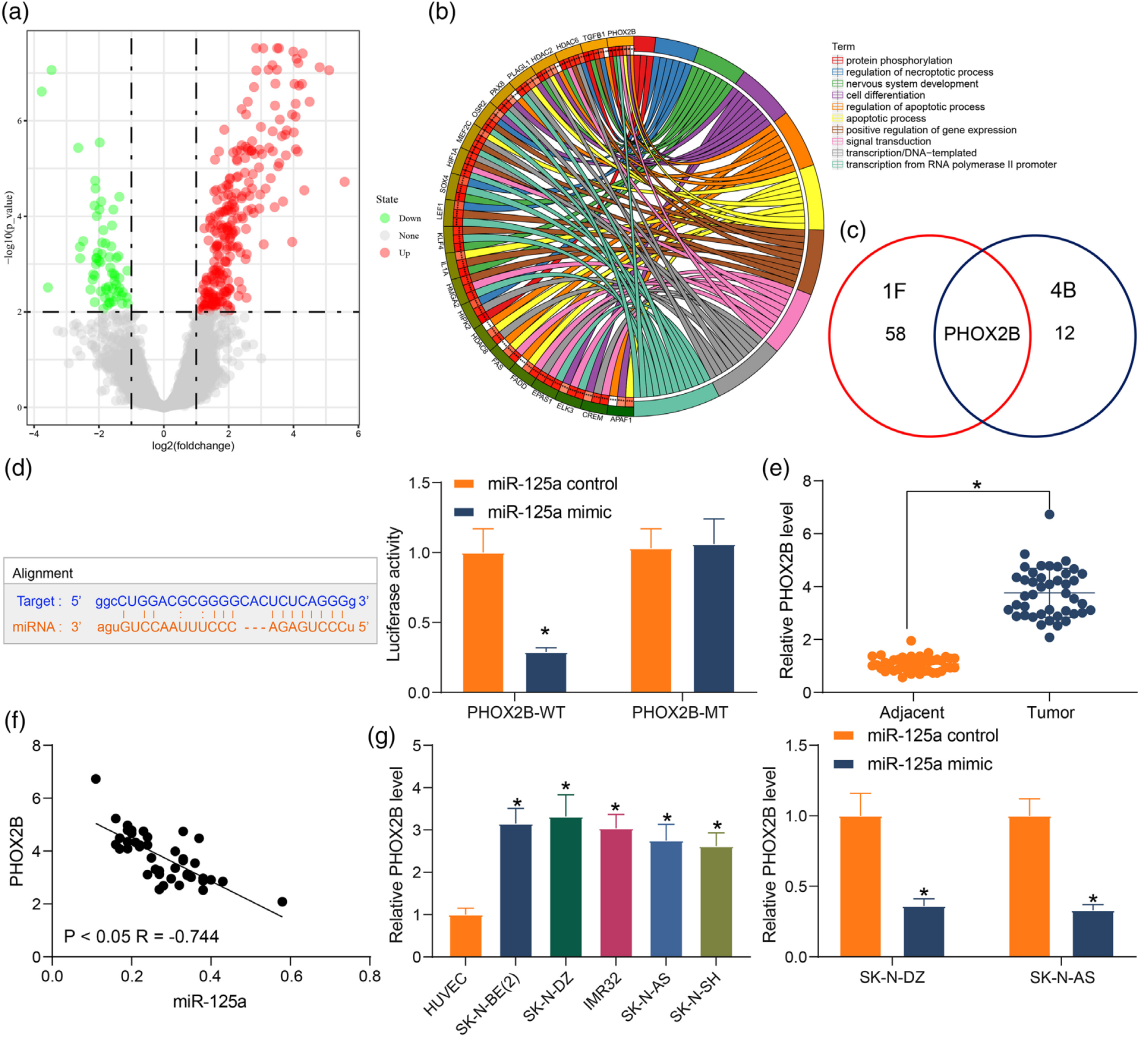
miR‐125a targets PAK6 and inhibits its expression. (a) The volcanic map for differentially expressed genes. (b) Gene Ontology (GO) enrichment analysis of differentially expressed genes. (c) miR‐125a downstream genes screened using a Venn map. (d) The targeting relationship between PHOX2B and miR‐125a examined using dual‐luciferase assay. (e) PHOX2B mRNA expression in neuroblastoma (NB) tissues by reverse transcription‐quantitative (RT‐q) PCR. (f) Pearson's analysis of the correlation between PHOX2B and miR‐125a in NB tissues. (g) mRNA expression of PHOX2B in NB cells and miR‐125a high expressing cells by RT‐qPCR. Results are shown as mean ± SD, representative of three technical replicates (*p < .05 vs. adjacent tissues, human umbilical vascular endothelial cell [HUVEC], or miR‐125a control by paired *t*‐test for panel E and by two‐way analysis of variance [ANOVA] for panels D and G)

According to the gene transcription analysis between NB patients and healthy young donors in the GEO database GSE25624, 120 genes were upregulated and 21 genes were downregulated in NB (Figure 4a). GO enrichment analysis of the differentially expressed genes revealed that 12 genes were mainly enriched in apoptosis and cell differentiation processes (Figure 4b). Since we obtained genes among miR‐125a target genes enriched in apoptosis and cell differentiation process in Figure 1f, we compared these genes with those in Figure 4b and found that the overlapping gene was PHOX2B (Figure 4c). We then suggested that PHOX2B might be the gene mediated by miR‐125a in NB. To further prove this hypothesis, we first verified the binding sites between miR‐125a and PHOX2B by a dual‐luciferase assay and found that miR‐125a mimic could indeed lead to a reduction in fluorescence intensity of PHOX2B‐WT plasmid (Figure 4d). The expression of PHOX2B in NB tissues was found to be significantly elevated (Figure 4e) and negatively correlated with miR‐125a expression (Figure 4f). Detection of PHOX2B expression in cells revealed that PHOX2B was highly expressed in NB cell lines and significantly reduced by miR‐125a induction (Figure 4g).

### 3.5 miR‐125a expression is mediated by HDAC2

**FIGURE 5:**
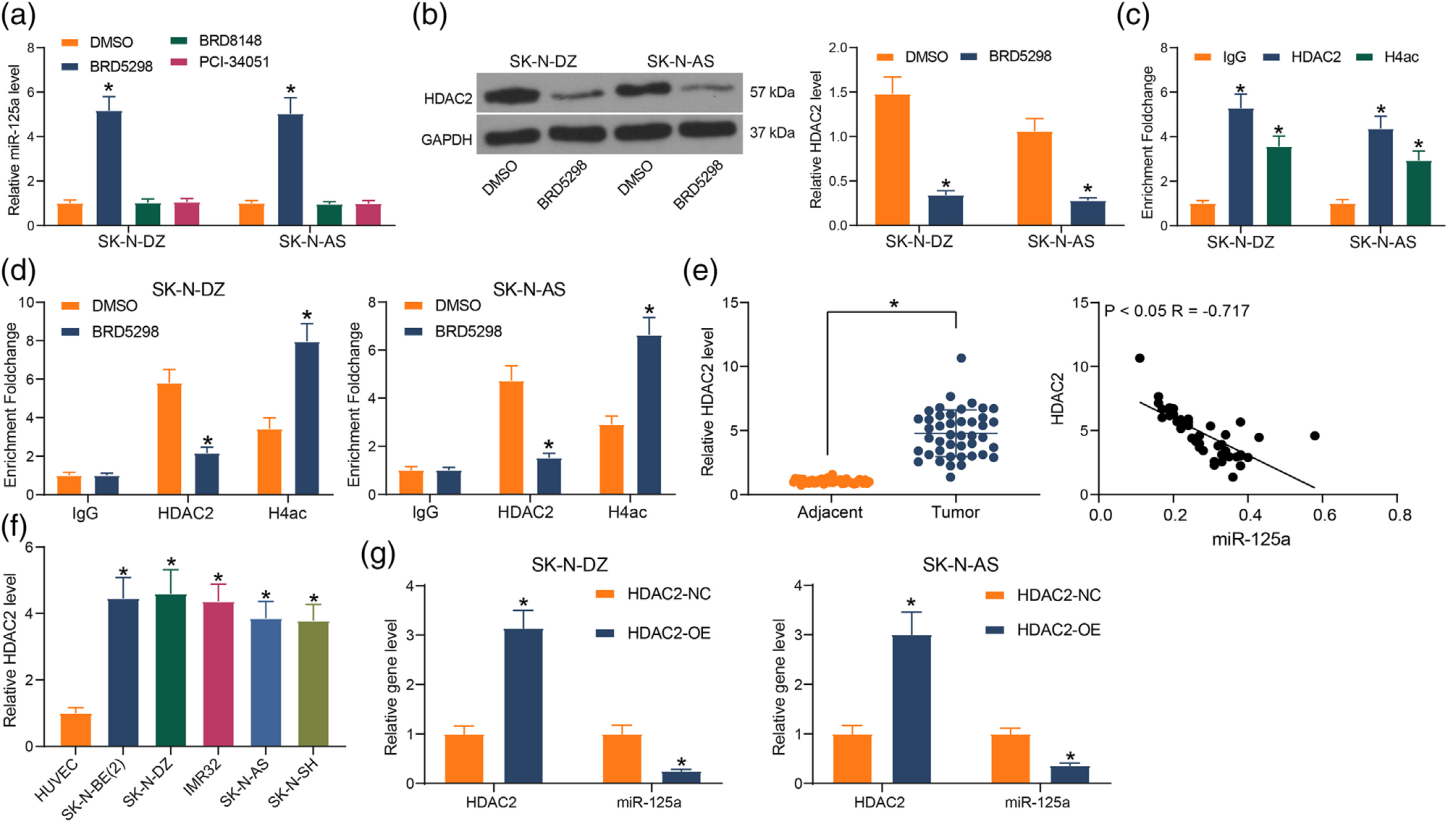
Histone deacetylase 2 (HDAC2) is the upstream gene for miR‐125a in neuroblastoma (NB). (a) Expression of miR‐125a in cells treated with the HDAC2 inhibitor BRD5298, the HDAC6 inhibitor BRD8148, and the HDAC8 inhibitor PCI‐34051 determined using reverse transcription‐quantitative (RT‐q) PCR. (b) HDAC2 mRNA expression in cells determined using RT‐qPCR assay. (c) The enrichment of HDAC2 with H4ac in the miR‐125a promoter examined using chromatin immunoprecipitation (ChIP) assay. (d) The enrichment of HDAC2 and H4ac in miR‐125a promoter after BRD5298 treatment determined using ChIP assay. (e) HDAC2 mRNA expression in NB tissue by RT‐qPCR and its correlation with miR‐125a. (f) HDAC2 mRNA expression in NB cells by RT‐qPCR. (g) HDAC2 mRNA and miR‐125a in cells in response to HDAC2‐OE examined using RT‐qPCR. Results are shown as mean ± SD, representative of three technical replicates (*p < .05 vs. DMSO, IgG, or HDAC2‐NC by paired *t*‐test for panel E, by one‐way analysis of variance [ANOVA] for panel F, and by two‐way ANOVA for panels A, B, C, D, and G)

After probing the downstream gene of miR‐125a, we revisited the results of Figure 4b and found an enrichment of HDACs in Figure 4c. We were curious whether epigenetic modifications play a role in mediating miR‐125a expression or NB cell activity. To explore the upstream gene of miR‐125a, we treated NB cells with specific HDAC inhibitors and later examined the miR‐125a expression in the cells in which only the HDAC2 inhibitor BRD5298 enhanced miR‐125a expression profile (Figure 5a). To verify the effect of the inhibitor, the expression of HDAC2 in the cells was significantly reduced, indicating that HDAC2 downregulation activated the expression of downstream miR‐125a (Figure 5b). Since HDAC2 represses gene transcription by mediating histone H4 deacetylation, the promoter of miR‐125a was obtained by UCSC. The detection of HDAC2 enrichment with H4 acetylation on the miR‐125a promoter in SK‐N‐DZ and SK‐N‐AS cells revealed that miR‐125a enrichment using antibodies to HDAC2 and H4ac was stronger than that using IgG (Figure 5c). Meanwhile, HDAC2 enrichment was decreased in SK‐N‐DZ and SK‐N‐AS cells treated with BRD5298, while H4ac enrichment was increased (Figure 5d). The expression of HDAC2 was detected in NB tissues, and HDAC2 was found to be significantly elevated in NB tissues. Correlation analysis between HDAC2 and miR‐125a showed a significant negative correlation (Figure 5e). Detection of HDAC2 expression in NB cells revealed that it was significantly higher in NB cells (Figure 5f). Using HDAC2‐OE to transfect NB cells, we observed that miR‐125a was significantly reduced (Figure 5g), indicating the transcriptional repression of miR‐125a by HDAC2 during the NB.

### 3.6 HDAC2/miR‐125a/PHOX2B affects NB activity in vitro

**FIGURE 6:**
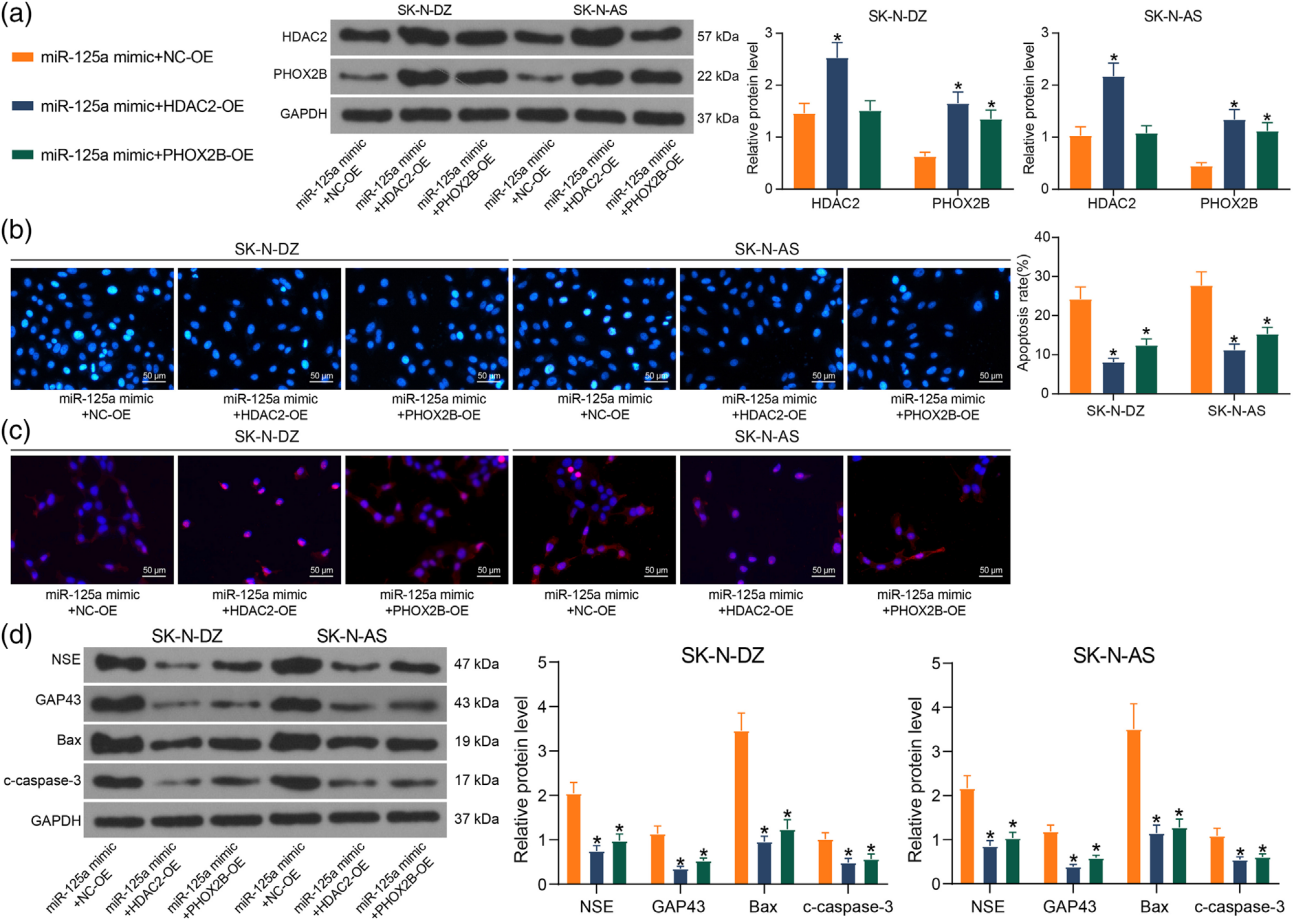
The histone deacetylase 2 (HDAC2)/miR‐125a/PHOX2B axis influences the apoptosis and differentiation of neuroblastoma (NB) cells in vitro. NB cell lines were cotransfected with miR‐125a mimic + HDAC2‐OE or miR‐125a mimic + PHOX2B‐OE. (a) HDAC2 and PHOX2B protein expression in cells was determined using western blot. (b) Cell apoptosis assessed by Hoechst staining. (c) Immunofluorescence detection of β‐Tubulin‐III expression in cells to assess cell differentiation. (d) Protein expression of GAP43, NSE, Bax, and cleaved‐caspase‐3 in cells examined using western blot. Results are shown as mean ± SD, representative of three technical replicates (*p < .05 vs. miR‐125a mimic + NC‐OE by two‐way analysis of variance [ANOVA])

To confirm whether PHOX2B is a key target of miR‐125a and miR‐125a is a downstream regulator of HDAC2, we tested whether ectopic expression of PHOX2B or HDAC2 could counteract the effect of miR‐125a on NB cellular activity. We transfected NB cells with fragments expressing HDAC2 and PHOX2B in miR‐125a overexpressing cells. Western blot assays of HDAC2 and PHOX2B protein expression in cells showed that both HDAC2‐OE and PHOX2B‐OE were successfully transfected (Figure 6a). The result of Hoechst staining showed that the pro‐apoptotic effect of miR‐125a on NB cells was partially neutralized by the overexpression of HDAC2 and PHOX2B (Figure 6b). The observation of cell morphology and differentiation demonstrated that the differentiation of cells under the influence of miR‐125a was inhibited by HDAC2 and PHOX2B, and the expression of β‐Tubulin‐III in cells was reduced (Figure 6c). Finally, the quantification of protein levels in cells revealed that the pro‐apoptotic protein expression was reduced under the influence of HDAC2, and the differentiation‐related protein expression was also diminished. Cells overexpressing PHOX2B showed the same trend as these overexpressing HDAC2 (Figure 6d). It was demonstrated that both HDAC2 and PHOX2B were able to inhibit the effect of miR‐125a to increase NB cell activity again.

### 3.7 HDAC2/miR‐125a/PHOX2B affects NB activity in vivo

**FIGURE 7:**
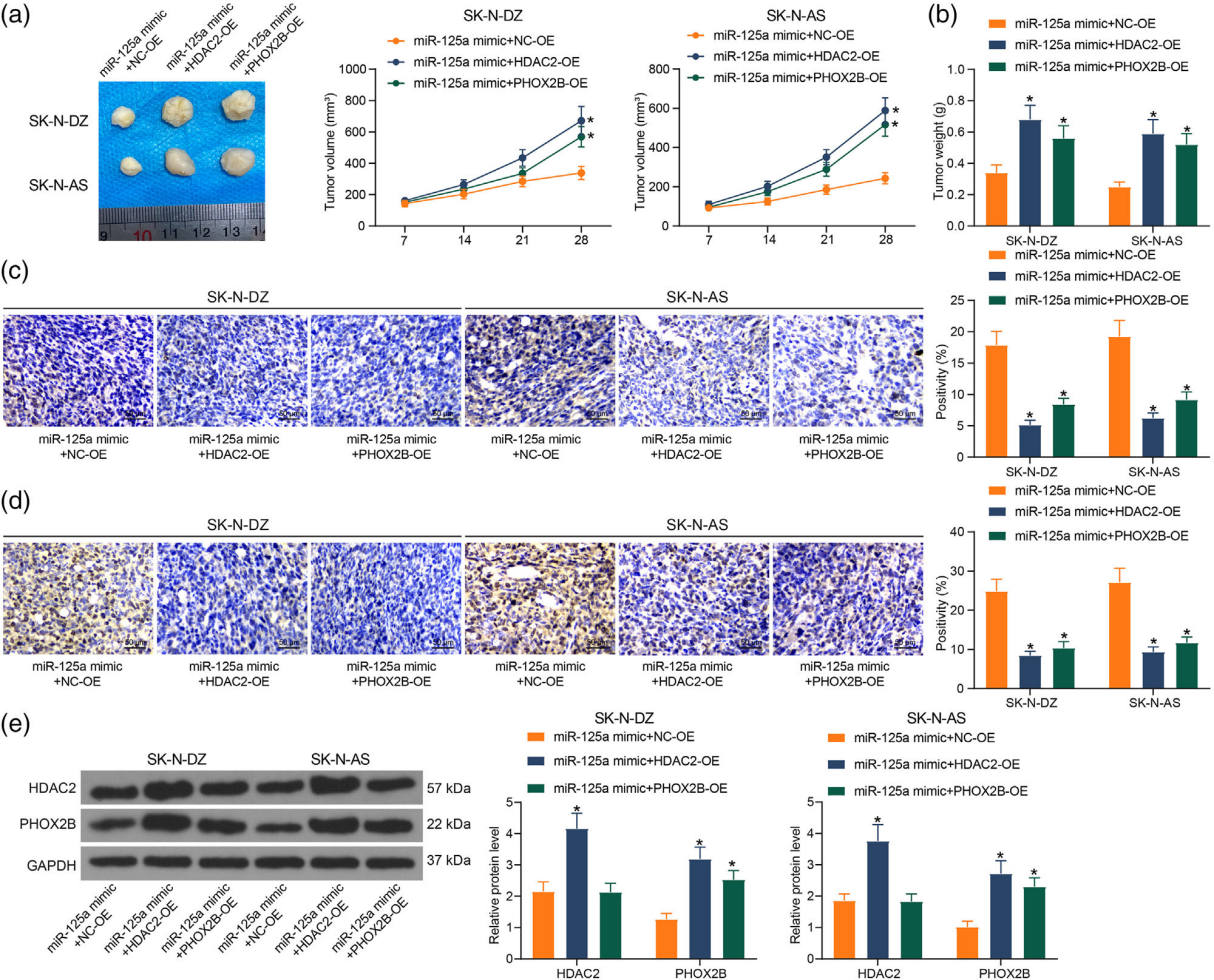
The histone deacetylase 2 (HDAC2)/miR‐125a/PHOX2B axis influences the proliferation of neuroblastoma (NB) cells in vivo. Nude mice were injected with stably transfected NB cells. (a) Tumor growth curve after injection of cells. (b) Assessment of tumorigenic activity of cells by tumor weight. (c) Immunohistochemical detection of changes in cleaved‐caspase‐3 levels in tumor tissues. (d) Immunohistochemical detection of changes in β‐Tubulin‐III levels in tumor tissues. (e) The HDAC2 and PHOX2B protein expression in tumor tissues by western blot. Results are shown as mean ± SD, representative of three technical replicates (*p < .05 vs. miR‐125a mimic + NC‐OE by two‐way analysis of variance [ANOVA])

In the last part, we set out to evaluate the functional effects of HDAC2, miR‐125a, and PHOX2B on the NB progression. The upregulation of HDAC2 and PHOX2B accelerated tumorigenesis and enhanced tumor volume after 28 days (Figure 7a). It revealed that upregulation of HDAC2 and PHOX2B promoted tumor weight (Figure 7b). Immunohistochemistry showed that apoptosis and differentiation in tissues were significantly reduced by HDAC2 and PHOX2B (Figure 7c,d). Finally, the protein expression of HDAC2 and PHOX2B in tissues was examined by western blot. It was noted that both HDAC2 and PHOX2B overexpression were consistently expressed in xenograft tumors after upregulation of HDAC2 and PHOX2B (Figure 7e), suggesting that HDAC2 mediates the PHOX2B expression through miR‐125a in vivo.

## 4 DISCUSSION

Significant progress has been achieved to understand the molecular mechanisms related to the etiology and pathogenesis of NB, and genome‐wide association investigation, transcriptomics, genome sequencing, and high‐throughput genome analysis have revealed genetic alterations and impaired pathways, which hold accountable for NB growth and development (Zafar et al., 2021). Moreover, the significance of miRNAs in the mediation of cell survival, proliferation and differentiation, and their involvement in the pathogenesis of NB has been appreciated in a recent review (Rezaei et al., 2021). In the present study, we found through microarray analysis that miR‐125a was downregulated in NB, which was associated with short survival of NB patients and resistance to cell apoptosis and differentiation. Specifically, miR‐125a overexpression induced NB cell apoptosis and differentiation but suppressed tumor growth in vivo. We also found that miR‐125a targeted and negatively regulated PHOX2B expression. Further, the downregulation of miR‐125a in NB cells was related to HDAC2 overexpression.

The expression of miR‐125a was downregulated in several types of cancers, including hepatocellular carcinoma (Potenza et al., 2017), lung cancer (Sun et al., 2020), gastric cancer (Xu et al., 2014), ovarian cancer (Lee et al., 2016), colon cancer (Tong et al., 2015), retinoblastoma (Zhang et al., 2016), and glioblastoma (Cortez et al., 2010). Moreover, in cervical cancer, miR‐125a high expression predicted favorable outcome for patients (Fan et al., 2015). Our analysis also found that miR‐125a was much lower in MNA patients than non‐MNA patients, suggesting the specificity of the prognostic role of miR‐125a in NB. miR‐125a‐5p overexpression enhanced the antiproliferative and pro‐apoptotic effects of gefitinib on the nasopharyngeal carcinoma cells (Liu et al., 2014). In addition, miR‐125a‐5p inhibited glioblastoma cell proliferation and elevated cell differentiation (Yuan et al., 2015). Consistently, we observed that miR‐125a overexpression enhanced the expression of Bax, cleaved‐caspase‐3, GAP43, and NSE in NB cells, indicating the supporting role of miR‐125a on NB cell apoptosis and differentiation. Cannabidiol was found to partially counteract the depletion of GAP43 and β‐Tubulin‐III, thus alleviating the injury of NB SH‐SY5Y cells (Branca et al., 2019). Also, increased apoptosis and cleaved‐caspase‐3 were observed in mouse granulosa cells transfected with a miR‐125a‐5p mimic (Wang et al., 2016). This was largely in agreement with our in vitro and in vivo results regarding the assessment of cleaved‐caspase‐3 expression.

Integrated bioinformatics prediction and dual‐luciferase assay revealed that PHOX2B is a putative target of miR‐125a in NB, while HDAC2 binds to miR‐125a to negatively regulate its expression. Thwin et al. (2020) revealed that PHOX2B was one of the core genes upregulated in bone marrow of patients with NB relative to the peripheral blood sample. Furthermore, PHOX2B expression in bone marrow aspirate could be a biomarker for NB patients at high risk and with poor response to treatment (El‐Shazly et al., 2019). However, its functional role in NB needs to be clarified. Interestingly, Bachetti et al. (2015) found that miR‐204 mediated downregulation of PHOX2B expression in NB cells posttranscriptionally. This suggests that PHOX2B, regulated by a certain miRNA, could involve in the apoptosis and differentiation in NB. Our rescue experiments validated this hypothesis.

Aberrant HDAC recruitment and expression and dysregulated H4ac have been described for tumor cells (Witt et al., 2009). In this study, it was noted that miR‐125a expression only responded to HDAC2 inhibitor among three different HDAC inhibitors. The ChIP assay further substantiated that inhibition of HDAC2 expedited the H4ac, thus upregulating the expression of miR‐125a. Overexpression of HDAC2 was highly correlated with high tumor grade, positive lymph node status, and dismal prognosis, and the HDAC inhibitor showed antitumor effects on breast cancer lines by mediation of miR‐182 (Shan et al., 2017). Suppression of HDAC8 increased the doxorubicin sensitivity of NB cells via upregulation of miR‐137 (Zhao et al., 2017), which highlights the involvement of HDAC in NB progression. Lodrini et al. (2013) reported that HDAC2 depletion enhanced promoter‐associated histone H4ac and the following miR‐183 expression, indicating epigenetic changes preceded transcriptional activation in NB. In addition, silencing of HDAC3 inhibited PAK6 expression by elevating miR‐27a, ultimately inhibiting neuronal apoptosis and accelerating the recovery of spinal cord injury (Zhou et al., 2020). In the present study, we found that HDAC2 counteracted the stimulative effects of miR‐125a on NB cell apoptosis and differentiation.

## 5 CONCLUSION

In conclusion, this study found that HDAC2 expression was significantly upregulated in NB cells, which inhibited miR‐125a and upregulated PHOX2B expression in an H4ac‐dependent manner. In addition, the study suggested that targeting miR‐125a may be a therapeutic candidate for NB patients. Other pathways of miR‐125a regulation will be explored in future studies.

## CONFLICT OF INTEREST

The authors declare no conflict of interest.

## Data Availability

All the data generated or analyzed during this study are included in this published article.
